# The Role of Reprojected Planar Data Derived from Fast Whole-Body Bone SPECT/CT

**DOI:** 10.3390/diagnostics16203434

**Published:** 2026-10-21

**Authors:** Mansour M. Alqahtani, Kathy Willowson, Chris Constable, Roger Fulton, Elizabeth Bailey, Sally Ayesa, Geoff Schembri, Peter L. Kench

**Affiliations:** 1 Department of Radiology and Medical Imaging, Faculty of Applied Medical Sciences, Najran University, Najran 1988, Saudi Arabia; 2 Discipline of Medical Imaging Science, Faculty of Medicine and Health, The University of Sydney, Sydney, NSW 2006, Australia; chris.constable@hermesmedical.com (C.C.); roger.fulton@sydney.edu.au (R.F.); peter.kench@sydney.edu.au (P.L.K.); 3 Health Research Centre, Najran University, Najran 1988, Saudi Arabia; 4 Institute of Medical Physics, Faculty of Science, The University of Sydney, Sydney, NSW 2006, Australia; 5 Department of Nuclear Medicine, Royal North Shore Hospital, St Leonards, NSW 2065, Australia; elizabeth.bailey2@health.nsw.gov.au (E.B.); sally.ayesa@sydney.edu.au (S.A.); geoffschembri@gmail.com (G.S.); 6 HERMES Medical Solutions, Strandbergsgatan 16, 112 51 Stockholm, Sweden; 7 Faculty of Medicine and Health, The University of Sydney, Sydney, NSW 2006, Australia

**Keywords:** SPECT/CT, fast acquisition time, reprojected planar image, bone imaging

## Abstract

**Background/Objectives:** The purpose of this prospective study was to investigate the preferred reconstruction SPECT parameters for generating reprojected planar images in bone studies, and to evaluate, by visual reader assessment, the feasibility of using these reprojected images as a substitute for traditional planar whole-body (WB) bone scan images. This was a single-centre feasibility study; diagnostic accuracy was not assessed. **Methods:** Patients referred for planar bone scintigraphy (PBS) and WB-SPECT/CT were prospectively enrolled. Reprojected planar bone scintigraphy (R-PBS) data were generated using forward projection of the three-bed SPECT/CT data. Three nuclear medicine (NM) specialists, blinded to the imaging reconstruction, evaluated both sets of images in a randomised order. The preferred reconstruction parameters for R-PBS were determined by visual assessment using a 4-point ordinal visual scoring scale. A side-by-side comparison of PBS vs. R-PBS was conducted, including assessments of image quality, artefact generation, image preference, and any clinical significance that may affect report interpretation. The agreement between the physicians’ evaluations was measured using the Kappa coefficient test. **Results**: Of the four reconstruction parameter combinations, 6 iterations, 8 subsets, and a 5 mm Gaussian post-filter yielded the highest visual quality scores. PBS images scored higher on image quality, though this difference was not statistically significant (*p* > 0.05). The inter-rater agreement for image quality scores was moderate for both PBS and R-PBS images. The results indicate that among the 33 reader-level assessments derived from 11 patient pairs and reviewed by the 3 readers, PBS images were preferred 13 times (~40%), R-PBS 10 times (30%), and no preference was indicated for the remaining 10 (30%). **Conclusions**: In this small feasibility cohort, R-PBS images derived from fast WB-SPECT/CT data were of visual quality comparable to PBS and may have potential as an alternative to PBS. If confirmed in larger studies with formal diagnostic accuracy assessment, R-PBS could remove the need for a separate PBS acquisition and reduce overall patient imaging time.

## 1. Introduction

Planar bone scintigraphy (PBS) has been the mainstay investigation for bone metastases (BM) and non-oncological bone diseases due to its low cost and ability to visualise the entire skeleton rapidly [1,2]. Over the years, the workflow for bone imaging has undergone gradual evolution. Initially, single field-of-view (FOV) “step and shoot” static imaging was performed over the entire skeleton. Subsequently, anterior and posterior whole-body planar images were acquired using continuous bed motion [3,4,5]. The sensitivity and accuracy of localisation were enhanced when technological advances in molecular imaging led to the development of hybrid tomographic imaging, specifically single-photon emission computed tomography with X-ray computed tomography (SPECT/CT). It provides excellent anatomical information that can improve sensitivity and specificity compared to PBS alone [6,7]. 

Continuous developments in SPECT/CT, a technique that has gradually moved from an “underutilised technological option” [8,9] towards a recognised role in strengthening the diagnostic capabilities of single-photon imaging, may have implications for patient management [8]. For example, a shorter SPECT acquisition may reduce the likelihood of patient motion and the overall study duration and, provided diagnostic quality is maintained, could facilitate the clinical use of whole-body (WB) SPECT/CT [10,11,12]. The emerging feasibility of standardised uptake value (SUV)-based quantification in SPECT/CT may contribute to longitudinal disease assessment, though standardisation remains an area of active investigation [13,14,15]. In nuclear medicine (NM) practice, these developments collectively support a transition from planar- to tomographic-first bone imaging workflows.

Beyond oncological bone imaging, SPECT/CT with technetium-99m-radiolabelled targeting vectors has shown emerging value in the early diagnosis and management of rheumatoid arthritis and degenerative bone diseases, where it may enable earlier detection of synovial, periarticular, and metabolic bone pathology than planar scintigraphy alone [16]. In parallel, hybrid PET/CT and SPECT/CT with quantitative and radiomic feature extraction are now considered the gold standard in many tumour-lesion assessments, not only in preclinical research, enabling quantitative imaging investigations that provide both morphological and functional information. In a representative preclinical example, PET imaging of Balb/c nude mice xenografted with a CCK2R-overexpressing A431 human epidermoid carcinoma cell line was processed, segmented after atlas-based co-registration, and 112 radiomic features were extracted for each organ/tissue examined, confirming targeted biodistribution of a novel ^68^Ga-labelled radiopharmaceutical [17]. The present study does not include quantitative or radiomic analysis. Instead, it addresses a narrower, complementary clinical question, namely whether the two-dimensional planar component of a bone scan can be derived from a fast three-dimensional WB-SPECT/CT acquisition without a dedicated planar scan.

Several authors have suggested that WB-SPECT/CT may eventually replace PBS [18,19]. Next-generation fixed-ring geometry SPECT cameras have been introduced that could enable WB tomographic imaging in a short acquisition time; however, they do not offer the ability to acquire 2D planar data due to their design [20,21]. Before planar data can be discarded, it will be important to demonstrate equivalence or superiority of generated planar data or the superiority of SPECT/CT data in isolation. While a reprojected planar image may be an ideal solution to facilitate the transition towards a routine WB-SPECT/CT bone scanning only [21]; however, NM specialists may reasonably be cautious about dispensing with proven data. When the change from planar to SPECT lung scanning occurred, there was initial reluctance and validated reprojected planar data was utilised to allow reporting specialists to slowly make the transition with confidence [22,23]. The acquisition of both PBS and WB-SPECT/CT consecutively during this transition period may be ideal though increases the examination duration, impacting patient comfort and workflow. Another option is to generate simulated planar images from the SPECT/CT data, which does not require an increase in scanning time and can therefore maintain patient throughput [23]. 

The use of reprojected “synthetic” planar images from SPECT data, particularly for lung imaging is well documented [22,23,24,25]. However, to the authors’ knowledge, only a limited number of studies have evaluated reprojected planar bone scintigraphy (R-PBS) acquisition in the investigation of bone scans. Moreover, these studies have focused on either a novel algorithm method (presented in conference proceedings) [3] or new camera types, such as cadmium-zinc-telluride (CZT) detectors [21]. Another prospective study reported the possibility of performing R-PBS; however, the total scanning examination time was 30 min, which increased the imaging time burden, and the R-PBS were generated from SPECT data acquired in list-mode format and subsequently resampled. Although the result revealed that R-PBS had excellent value to facilitate the reading of SPECT/CT, the reconstruction algorithm parameters for R-PBS were not investigated [26]. We have previously demonstrated that the use of fast WB-SPECT/CT in place of a standard SPECT/CT protocol is feasible for bone scans, while maintaining image quality [10,27].

Forward projection of SPECT-reconstructed three-dimensional (3D) data into reprojected anterior/posterior 2D projections may therefore allow planar images to be displayed without a separate planar acquisition [22,23]. Keeping 2D images available in this way could help clinicians during any transition towards 3D-only reporting, given their historical familiarity with planar images. The present work is a single-centre, prospective feasibility study based on visual (qualitative) assessment by experienced readers. Its aims were (i) to identify, among four clinically guided SPECT reconstruction parameter sets, the preferred set for generating R-PBS; and (ii) to compare R-PBS with conventionally acquired PBS in terms of reader-rated image quality, artefacts, image preference and clinically significant discrepancies, together with inter-reader agreement. Objective quantitative image-quality metrics and lesion-level diagnostic accuracy were not assessed. The study is therefore intended as a first step to determine whether R-PBS warrants formal diagnostic evaluation as a potential substitute for acquired PBS.

## 2. Materials and Methods

### 2.1. Patient Selection

This prospective study was conducted at Royal North Shore Hospital, Sydney, Australia. Planar bone scintigraphy and WB-SPECT/CT imaging were performed on 17 patients enrolled in a prospective study. The patients who were included in this study were referred as part of a routine clinical WB bone imaging investigation. The clinical indications included a variety of oncological, infection, and/or chronic diseases. 

Six patients were excluded due to incomplete data, leaving 11 patients for analysis (Phase 2: image comparison cohort). For Phase 1 (reconstruction parameter evaluation), eight patients were randomly selected from this cohort. To determine the best-performing SPECT reconstruction parameters for R-PBS generation, each patient’s SPECT data were reconstructed using four different parameter sets, yielding a total of 32 reconstructed images. The 32 R-PBS images from Phase 1 were independently reviewed by three NM specialists. All data were anonymised prior to analysis.

The Northern Sydney Local Health District Human Research Ethics Committee approved the study on 22 February 2021, with reference number 2020/ETH02150. Patient consent was waived because the study was low risk and used data collected during routine clinical care.

### 2.2. Bone Scintigraphy

#### Planar Bone Scintigraphy

Standard PBS (anterior and posterior projection images of the entire skeleton) images were acquired 2–3 h after injection of ^99m^Tc-labelled hydroxymethylene diphosphonate (^99m^Tc-HDP), with a dose of 845 ± 21 (mean ± SD) MBq in accordance with the EANM and SNMMI procedure guidelines for bone scintigraphy [5,28]. Participants were orally hydrated and emptied their bladders before the scan. All imaging data were acquired using a Symbia dual-head gamma camera (Symbia T16 TruePoint, Siemens Healthineers, Erlangen, Germany) equipped with low-energy high-resolution (LEHR) collimators. The scan acquisition time of the PBS was 15–20 min, with a matrix size of 256 × 1024, and a zoom factor of 1 and a 15% energy window that was centred on the ^99m^Tc photopeak at 140 keV.

### 2.3. Whole-Body SPECT/CT 

Following WB PBS, a three-bed WB-SPECT from skull vertex to the mid-thigh was acquired using the following parameters: 8 s per projection, 60 projections per detector head (120 projections in total) over 360° rotation in a continuous non-circular contoured orbit. The three-bed WB-SPECT/CT acquisition protocol follows our previous work on fast WB-SPECT/CT [10,27] and is consistent with similar protocols reported for CZT and conventional systems [29,30]. Imaging below the thighs was excluded for the purposes of this study to reduce time imposition on patients. The SPECT data were acquired using a 128 × 128 matrix size with a 4.8 mm pixel size. In all cases, a low-dose CT scan was performed after SPECT imaging, for anatomical localisation and attenuation correction purposes, utilising beam parameters of 110 kV, 50 mAs with adaptive dose modulation (CARE Dose 4D, Siemens Medical Solutions) [12]. The total acquisition time of SPECT/CT scanning was approximately 24 min.

### 2.4. SPECT Reconstruction and Reprojection of Planar Bone Images

All SPECT/CT hybrid images were reconstructed using standard site clinical parameters: 3D Ordered Subset Expectation Maximisation (OSEM) algorithm (4 iterations, 8 subsets), and the subsequent application of a 3D Gaussian filter of 8 mm full width at half maximum (FWHM) [31]. This algorithm applies resolution recovery using a distance-dependent 3D Gaussian collimator-detector model, attenuation correction (AC) based on a CT-derived linear attenuation map, and scatter correction (SC) based on the dual energy window approach [32]. 

Further reconstructions were performed using variations in the number of iterations (i), subsets (s), and Gaussian filter widths (FWHM) as summarised in Table 1. These four parameter sets were chosen empirically, guided by the current clinical protocol. The effect of these parameters on the image quality of the R-PBS data was examined, with the aim of finding the preferred reconstruction algorithm parameters for R-PBS.

**Figure 1:**
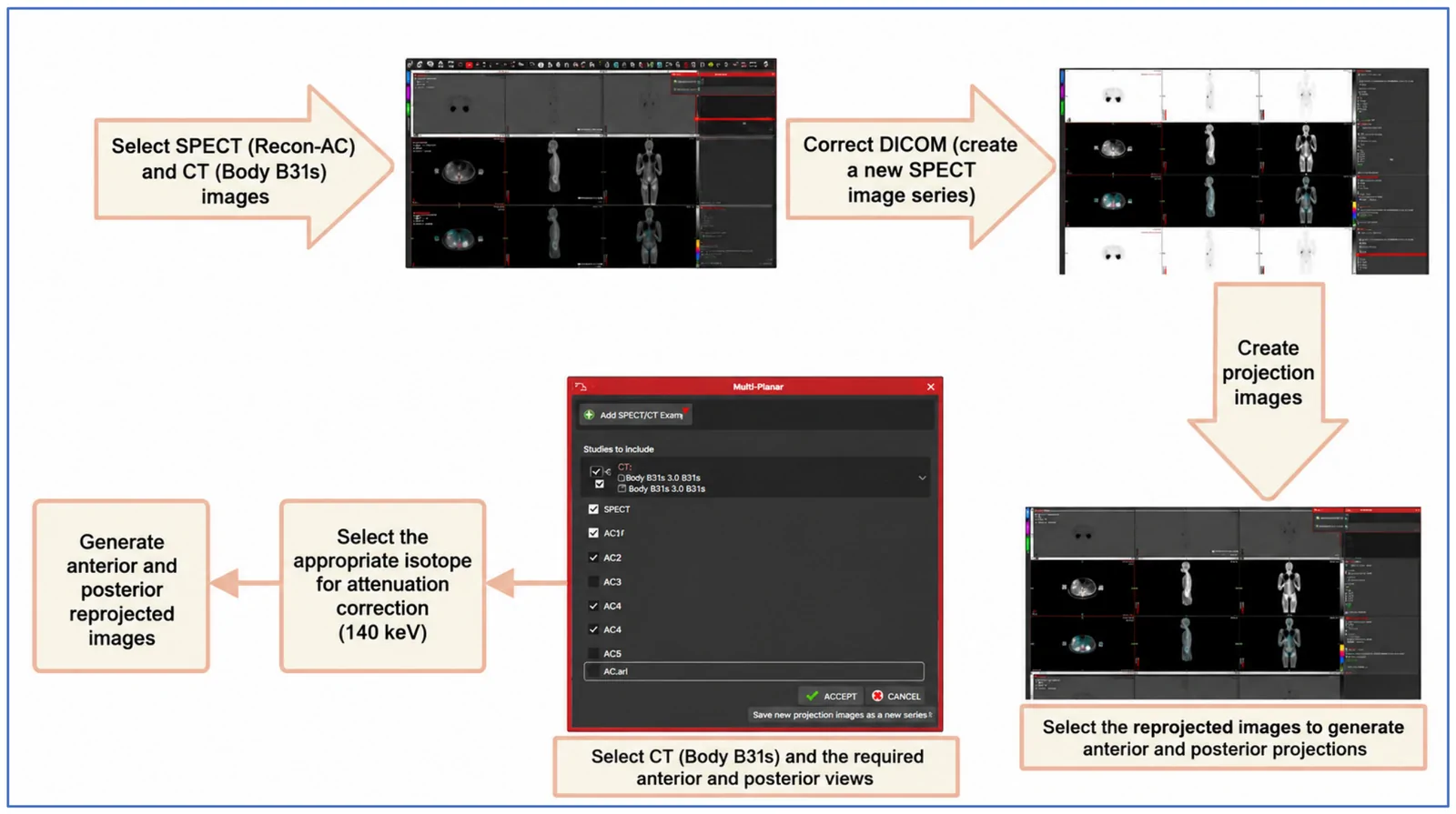
The process for generating R-PBS in MIM software.

Reprojected planar data were generated from SPECT reconstructions using forward projection. The R-PBS were generated using MIM (MIM Software Inc., Cleveland, OH, USA). Anterior and posterior reprojected planar images were generated by forward projecting the reconstructed 3D activity distribution through the CT-derived linear attenuation coefficient map, simulating the attenuation of emitted photons along each projection ray [26,33,34]. However, scatter correction and depth-dependent resolution modelling were not included in the forward projection process, resulting in R-PBS images that appear sharper and with higher contrast compared to conventionally acquired PBS images. Post-acquisition processing required an average of 1–2 min. The results were evaluated by the interpreting NM specialist physicians in comparison to the conventionally acquired planar bone scintigraphy (PBS) data. A diagram depicting the process for generating R-PBS is shown in Figure 1. 

### 2.5. Visual Assessment

In this study, patients underwent both WB-PBS and WB-SPECT/CT to generate R-PBS images for comparison. The visual assessment was conducted in two phases.

### 2.6. Phase 1: Reconstruction Parameter Evaluation

To identify the best-performing reconstruction parameters among the evaluated protocols for R-PBS generation, three NM specialist physicians, each with more than five years of experience in NM image interpretation, independently and blindly evaluated overall image quality using a 4-point ordinal visual scoring scale (1 = unacceptable, 2 = acceptable, 3 = good, 4 = excellent). This scale has previously been applied in bone imaging reader studies conducted by our group and others [26,27]. Eight patients were randomly selected from the cohort of 11, with each patient’s data reconstructed using four different combinations of iterations, subsets, and Gaussian filter widths as described above, yielding a total of 32 images. These images were randomly ordered and presented to each reader independently, without revealing the reconstruction parameters used. 

### 2.7. Phase 2: PBS vs. R-PBS Comparison

Following determination of the preferred reconstruction parameters, a pair of PBS and R-PBS images per patient (*n* = 11) were anonymised and independently evaluated by the same three NM specialist physicians. Readers were blinded to patients’ clinical history and other imaging investigations throughout. Each reader assessed all images, yielding 33 reader-level paired assessments.

The evaluation was conducted in two stages. In the first stage, the field of view (FOV) for all images was standardised to prevent readers from identifying whether images were PBS or R-PBS based on FOV differences alone. Readers attributed a score using the same 4-point ordinal visual scoring scale to assess overall image quality. In the second stage, the full FOV was revealed to enable readers to respond to image-type-specific questions.

All images were assessed across the following four domains:

**Overall image quality:** Readers were asked to rate the overall image quality using the 4-point ordinal visual scoring scale (1 = unacceptable, 2 = acceptable, 3 = good, 4 = excellent);

**Artefacts:** Readers were asked to indicate whether any obvious artefacts were present, and if so, to specify their location and type;

**Image preference:** Readers were asked to select their preferred image from each pair—PBS, R-PBS, or no preference—and to provide a reason for their selection, including factors such as lesion conspicuity, number of lesions identified, anatomical localisation, and diagnostic confidence;

**Clinical significance:** After the full FOV was revealed, readers were asked to identify any clinically significant findings visible on one image type that were not apparent on the other, and to indicate the direction of any such discrepancy.

### 2.8. Statistical Analysis

To assess inter-rater agreement in visual assessments, inter-rater agreement was calculated using Fleiss’ kappa (*k*) [35]. Cohen’s kappa (*k*) was also used to assess the agreement between the three sets of paired readers (Reader 1 vs. 2, Reader 1 vs. 3, Reader 2 vs. 3) [36]. The magnitude of agreement was categorised as slight (*k* = 0.00–0.20), fair (0.21–0.40), moderate (0.41–0.60), substantial (0.61–0.80), and almost perfect agreement (0.81–1.00) [37,38]. Kappa values were reported with corresponding 95% confidence intervals (95% CI). A Wilcoxon signed-rank test was conducted to determine whether statistically significant differences existed in image quality scores between PBS and R-PBS, applied separately for each reader. This nonparametric test was used because the data were not normally distributed, as determined using the Shapiro–Wilk test. The level of significance was set at *p* < 0.05. The statistical analyses were performed using jamovi software (version 2.3.0; The jamovi project) [39], an open-source R-based platform.

## 3. Results

### 3.1. Participant Demographics

The demographic and clinical characteristics of the 11 participants included in the analysis are summarised in Table 2. The cohort comprised six males and five females, with a median age of 74 years (range 55–89 years).

### 3.2. Image Quality Assessment

Overall image quality: preferred reconstruction parameters for reprojected planar bone images.

**Figure 2:**
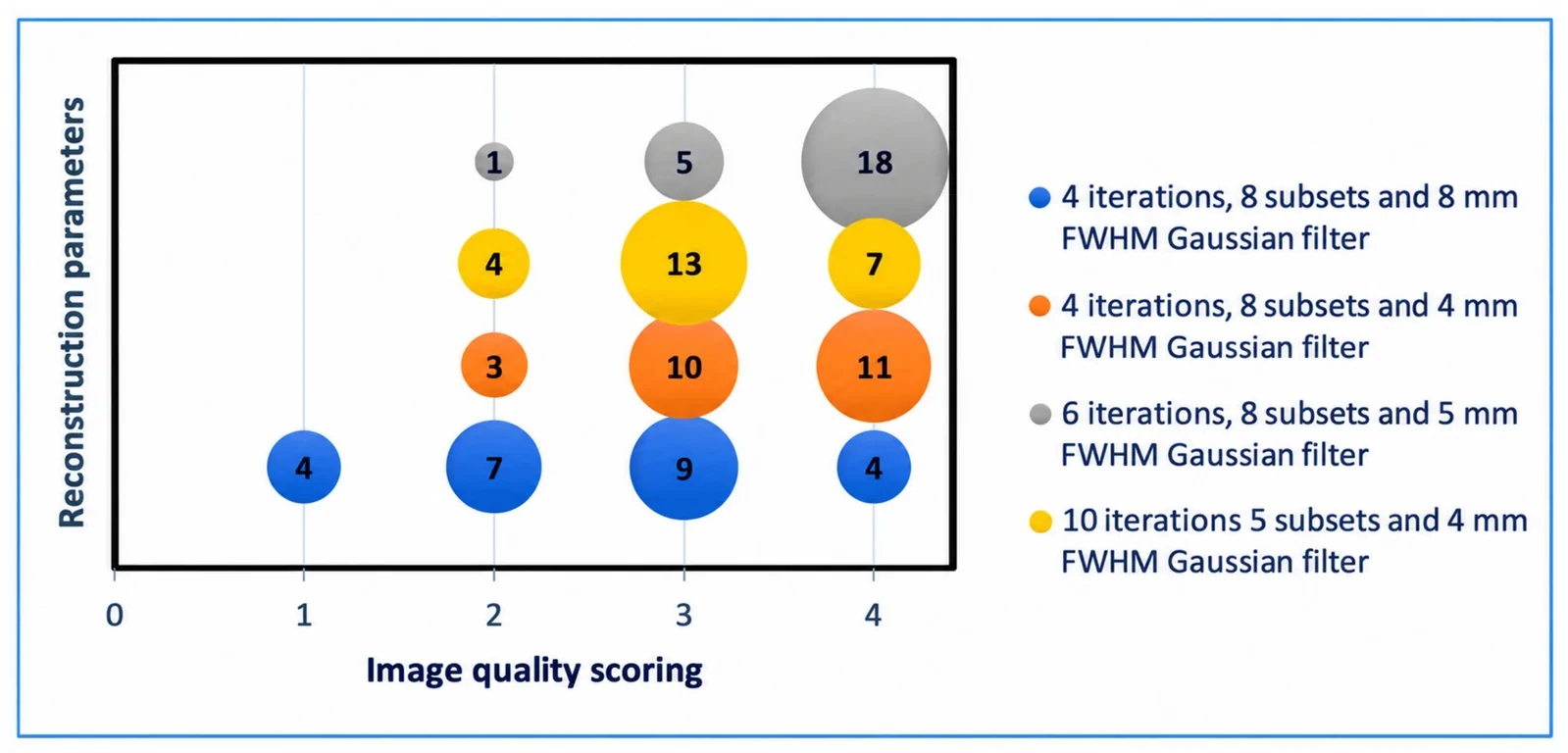
Bubble chart showing the image quality scores for four reconstruction parameters of R-PBS of bone patient studies, rated on a 4-point ordinal visual scoring scale ranging from 1 (unacceptable) to 4 (excellent) by three readers. The size of each bubble in the chart represents the frequency of a particular image quality score indicated within each bubble.

The readers were asked to evaluate the overall image quality of R-PBS using a 4-point visual scoring scale (1 = unacceptable, 2 = acceptable, 3 = good, 4 = excellent). A bubble chart was used to demonstrate the image quality scores for each reconstruction method scored by three readers, which can be seen in Figure 2. Each reconstruction review set contained 8 images, which were scored by three readers for a total of 24 images. 

Among the four reconstruction parameter combinations evaluated, the reconstruction using 6 iterations, 8 subsets, and a 5 mm Gaussian filter achieved the highest proportion of good-to-excellent image quality scores and was therefore selected for subsequent analyses (Table 3).

The inter-rater agreement between three readers was calculated to be k = 0.36 (95% CI 0.22 to 0.50), *p* < 0.001, which is considered fair agreement. However, the inter-rater agreement was substantial between readers 1 and 2, k = 0.69 (95% CI 0.45 to 0.86), *p* < 0.001. Therefore, the level of agreement between three readers was not higher than fair because reader 3 gave a lower image quality score than others. This inter-reader variability in scoring is reflected in the median values presented in Table 4.

**Figure 3:**
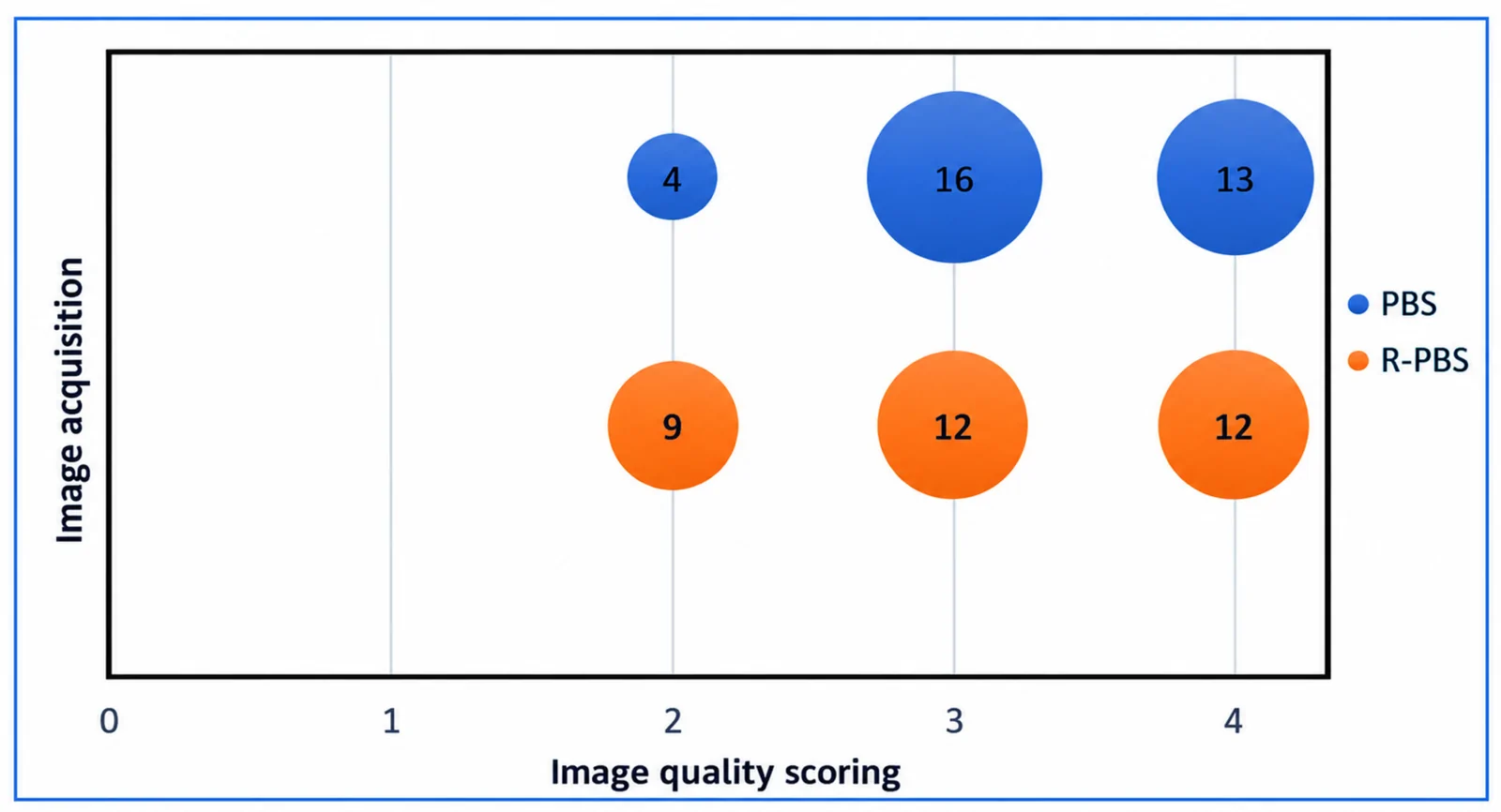
Bubble chart showing the image quality scores for PBS and R-PBS, rated on a 4-point ordinal visual scoring scale ranging from 1 (unacceptable) to 4 (excellent) by three readers. The size of each bubble in the chart represents the frequency of a particular image quality score. The specific frequency is indicated within each bubble.

**Figure 4:**
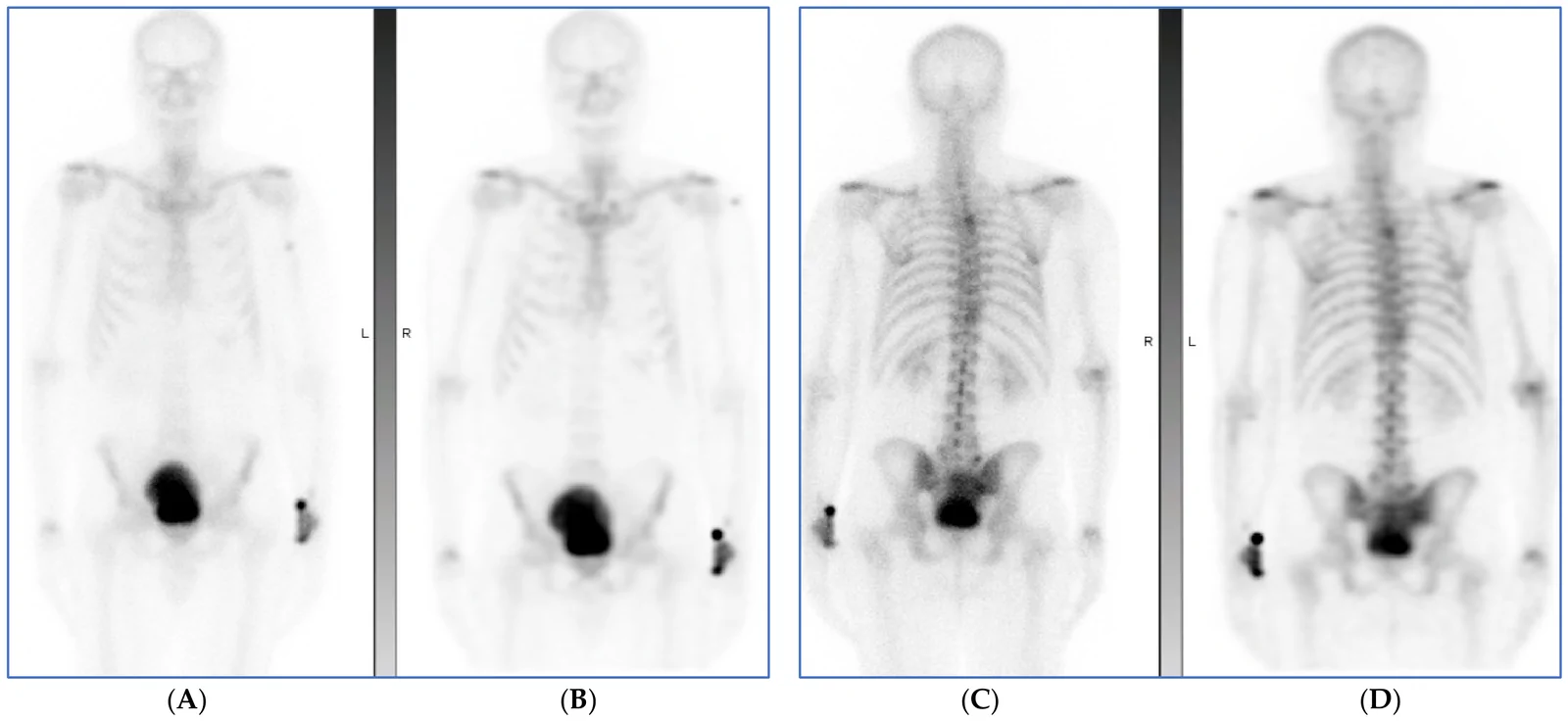
Shows the anterior (**A**) and posterior (**C**) views of PBS and anterior (**B**) and posterior (**D**) of R-PBS. The image quality of both acquisition protocols received a rating of 4 from all readers.

A bubble chart was used to demonstrate the image quality scores for PBS and R-PBS, which can be seen in Figure 3. The 33 reader-level paired assessments were derived from 11 patients and independently evaluated by three readers. The image quality score for the PBS and R-PBS images showed that 88% and 73% of the images were rated as good or excellent (scores of 3 or 4), respectively. However, only 12% and 27% were rated as acceptable image quality for PBS and R-PBS images, respectively. Moreover, it is also observed that no images scored as having unacceptable image quality for both PBS and R-PBS. The results of the pairwise Wilcoxon signed-ranks tests indicated that there were no statistically significant differences in image quality scores between PBS and R-PBS as assessed by each reader (Table 5). Examples of PBS and R-PBS images are shown in Figure 4.

The inter-rater agreement for image quality scores among readers for PBS and R-PBS images was found to be moderate, as indicated by a k value of 0.48 (95% CI 0.30 to 0.65), *p* < 0.001. The inter-rater agreement for image quality scoring was also moderate when calculated across pairs of readers (1 and 2, 1 and 3, and 2 and 3), with kappa values of 0.44, 0.57, and 0.48, respectively, all *p* < 0.001. The results of the image quality assessment for both PBS and R-PBS, as rated by each pair of readers, are presented in Table 6, Table 7 and Table 8.

### 3.3. Artefact Presentation

The readers evaluated whether there were any artefacts present in particular areas of the body in the PBS and R-PBS images. Across three readers, 76% of the PBS images were reported as having no artefact, while 24% were reported as having artefact. In contrast, 33% of the R-PBS images were reported as having no artefact, while 67% were reported as having an artefact (Table 9).

### 3.4. Reader Images’ Preference

**Figure 5:**
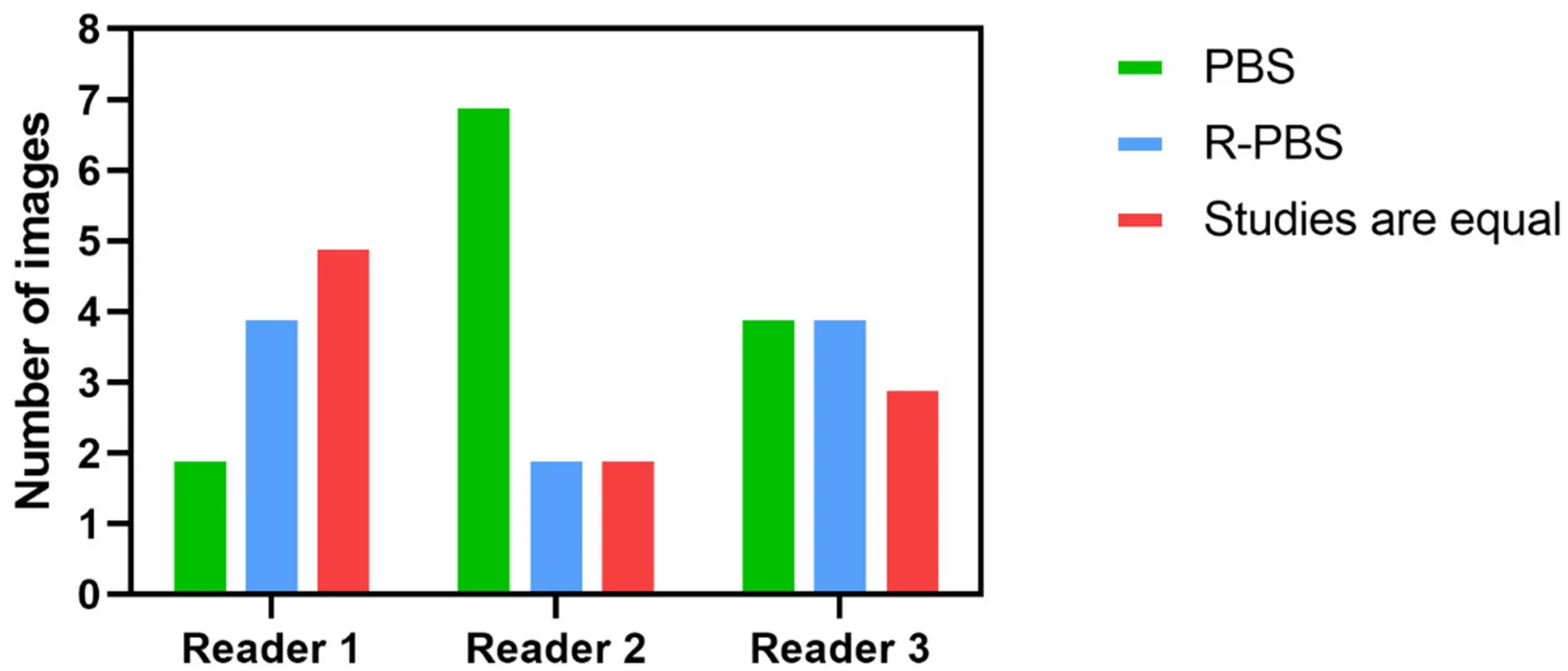
Bar plots showing the preferred images for each reader.

Here, readers were asked to select the most desirable images based on the superiority of the lesion to background, the number of lesion identifiers, the localisation, and the degree of confidence. Overall, readers preferred PBS images in 13 out of 33 images (~40%) and R-PBS images in 10 out of 33 cases (30%). In the remaining 10 out of 33 images (30%), readers selected the “studies are equal” category. The results of their preferences are shown in Figure 5, and the reasoning behind their choices is provided in Table 10. 

The most frequent reason selected for preferring PBS images was ‘increase the degree of confidence’. However, for the R-PBS images, the most frequent reason was ‘superior lesion to background’ contrast.

## 4. Discussion

This prospective study was conducted to determine the best-performing reconstruction parameters for R-PBS that is generated through the forward projection of three-bed SPECT data. Comparisons of the image quality of PBS and R-PBS were also made, with the aim of evaluating the utility of using R-PBS in clinical practice and determining whether R-PBS could potentially be used as a replacement for PBS images. To the best of our knowledge, this is the first study to systematically evaluate reconstruction parameters for R-PBS generation and to assess inter-rater agreement in visual image quality comparison between PBS and R-PBS by three independent readers. Very few studies on R-PBS have been conducted to date. Bandi et al. [3] presented a novel algorithmic approach in conference proceedings. Melki et al. [21] implemented the R-PBS method using a CZT camera. Arvola et al. [26] compared the diagnostic performance of PBS and R-PBS in the detection of bone metastases using multimodal imaging.

In recent years, significant progress has been made in the development of WB-SPECT/CT image techniques [40,41,42]. Software algorithms have played a crucial role in the formation and enhancement of SPECT/CT images [41,43]. Several vendor-neutral software programmes, such as Hermes Medical Solutions AB (Stockholm, Sweden) and Interactive Data Language (NV5 Geospatial Solutions, Boulder, CO, USA), have been utilised to facilitate the generation of R-PBS [23,25,26]. In this study, we used a MIM software (MIM Software Inc., Cleveland, OH, USA) to generate R-PBS images from SPECT/CT data. Our findings are consistent with recent evidence that routine bone SPECT/CT can be acquired at substantially reduced times while preserving image quality, noise characteristics, and diagnostic reliability; this has been demonstrated in both single-centre image-quality cohorts [12,27] and in subsequent CZT-based prospective studies [30,44]. The broader utility of fast-acquisition SPECT extends to dynamic functional imaging: recent evidence from dynamic SPECT (DynSPECT) assessment of hepatic function has shown that quantitative SPECT metrics correlate meaningfully with laboratory tests, the Child–Pugh score, MELD, and the histological activity index [45]. Future work should involve assessing R-PBS generation using multiple vendor-neutral software programmes and validating its diagnostic performance against both PBS and PET-based reference standards.

In the visual assessment of R-PBS reconstruction parameters, it was found that using 6 iterations, 8 subsets, and a 5 mm Gaussian filter resulted in the highest scores compared to the other three reconstruction parameters (Table 3 and Table 4; Figure 2). The image quality for images reconstructed with 4 iterations, 8 subsets, and an 8 mm Gaussian filter was the lowest, as rated by three readers. This was the only reconstruction that resulted in four images being reported as having unacceptable image quality. It also had the lowest number of images rated as good or excellent compared to the other three reconstruction parameters. Readers noted in their free-text comments that 17% of the images reconstructed with 4 iterations, 8 subsets, and an 8 mm Gaussian filter appeared “poor” or “blurry,” while no similar comments were made for the other reconstructions. Nevertheless, images reconstructed with 4 iterations, 8 subsets, and a 4 mm Gaussian filter, as well as 10 iterations, 5 subsets, and a 4 mm Gaussian filter, had similar scores and were slightly lower than images reconstructed with 6 iterations, 8 subsets, and a 5 mm Gaussian filter. Therefore, these findings suggest that the preferred reconstruction parameters for R-PBS, using the four clinical imaging protocol reconstruction parameters evaluated, are 6 iterations, 8 subsets, and a 5 mm Gaussian filter (Table 3 and Table 4).

The results of our study showed that the reading physician rated the image quality of PBS as higher than that of R-PBS, as demonstrated in Figure 3, which is in accordance with previous studies [26]. No statistically significant differences were observed in image quality scores between PBS and R-PBS for any reader (Reader 1: *p* = 1.00; Reader 2: *p* = 0.109; Reader 3: *p* = 0.727; Table 5). In addition, none of the images obtained using either method was deemed “unacceptable” image quality. It is important to note that the readers who evaluated the images were experienced in interpreting PBS but had no prior experience with R-PBS images. This lack of familiarity may have affected the image quality rating for R-PBS. It is speculated that these ratings will improve as the readers gain more experience with R-PBS images. It is also worth noting that the additional information provided by the 3D SPECT/CT was not taken into account in this study, yet would be readily available in clinical practice if R-PBS were being employed. The pattern of slightly higher PBS scores without a statistically significant difference is consistent with the findings of Arvola et al. [44] for reprojected planar bone imaging on a CZT system, and with earlier reprojected-planar reader studies in pulmonary ventilation/perfusion imaging where reprojected planar images performed similarly to traditional planar scans [23,24]. A note of caution is warranted: Le Roux et al. [25] reported that reprojected planar V/Q images were not a reliable substitute for traditional planar V/Q in the diagnosis of pulmonary embolism, which indicates that the equivalence of reprojected to acquired planar images is clinical-task-dependent and must be re-established for each diagnostic question.

The inter-rater agreement for the R-PBS assessment among the three readers was classified as fair according to the Fleiss’ k coefficient (0.36). However, Readers 1 and 2 made almost identical median image quality scores for four different reconstructed images, indicating a substantial level of agreement as determined by Cohen’s k coefficient (0.69) between the two readers, with a discordance rate of 19%. In addition, the agreement between PBS and R-PBS images was moderate, with discordance rates of 36% between readers 1 and 2, 27% between readers 1 and 3, and 36% between readers 2 and 3. We are not aware of similar studies on reader agreement for the visual assessment of bone imaging quality between PBS and R-PBS. Although Le Roux et al. [25] studied pulmonary embolism rather than bone disease, their finding of moderate inter-rater agreement between planar and reprojected images is broadly consistent with our results. A recent study by Arvola et al. [26] found substantial agreement in the diagnostic interpretation of the detection of BM in breast and prostate carcinoma. Kappa values can be influenced by the prevalence of ratings within each category—when ratings cluster heavily in one category, agreement by chance increases, which may reduce the kappa value even when observed agreement is relatively high [46]. Therefore, the independent ratings were conducted by having each reader assess image quality without knowledge or influence from the other reader’s assessment.

**Figure 6:**
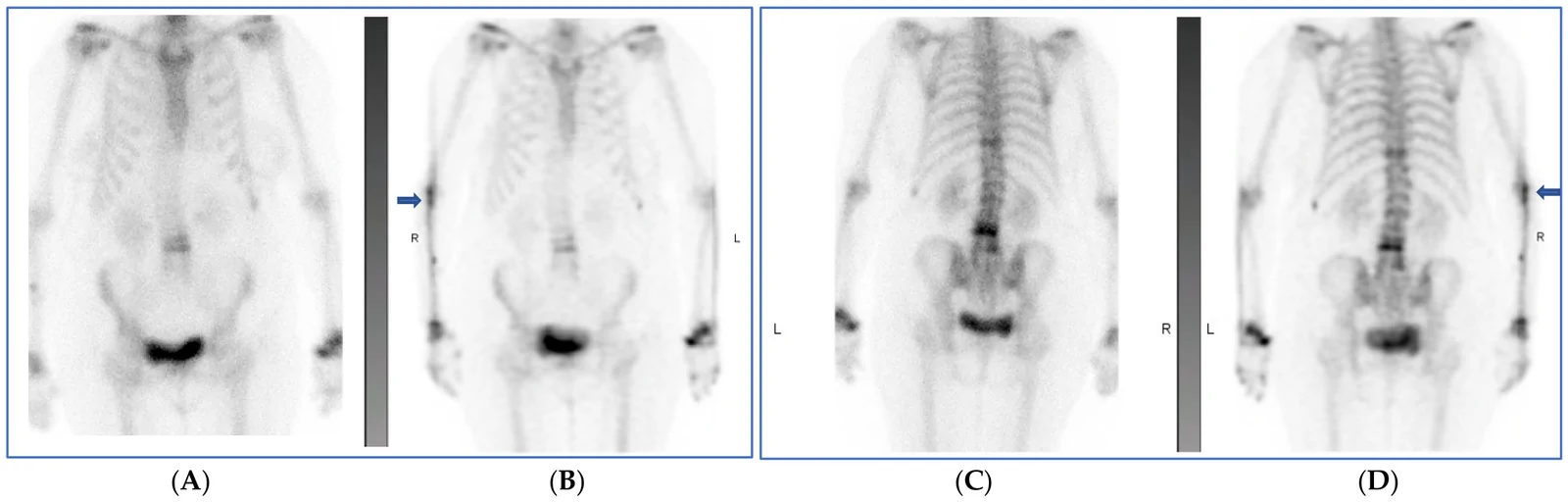
Shows the anterior (**A**) and posterior (**C**) views of PBS and anterior (**B**) and posterior (**D**) of R-PBS images. Anterior and posterior of R-PBS images show the presence of artefacts. Arrows indicate the artefacts.

**Figure 7:**
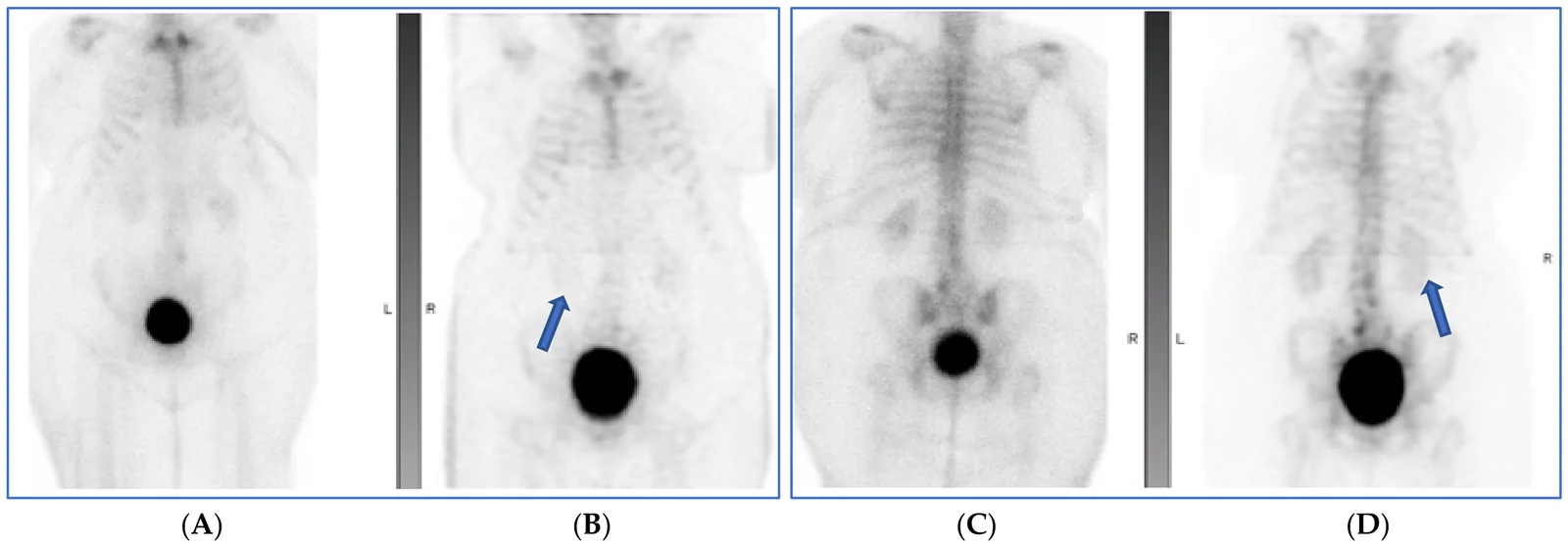
Shows the anterior (**A**) and posterior (**C**) views of PBS and anterior (**B**) and posterior (**D**) of R-PBS images. Both acquisition methods show stitch artefacts, although their presence is more pronounced in the R-PBS images. Arrows indicate the stitch artefacts.

The number of artefacts observed in the R-PBS images was higher than in the PBS images, as shown in Table 9. The artefacts present in the PBS images were primarily characterised as soft tissue attenuation artefacts and mild stitch artefacts. In contrast, the R-PBS images contained additional types of artefacts, including edge or truncation artefacts and a straight line crossing the midline of the body, resulting in a stitch artefact. Some readers suggested that truncation of the CT field of view (FOV), which is a recognised source of reconstruction artefacts [47], or the injection site may have contributed to the observed findings, particularly as the readers were blinded to other imaging investigations (Figure 6). Stitch artefacts were particularly pronounced in the abdominal area of R-PBS images, which may be due to the body habitus of the patient and more soft tissue uptake (Figure 7). One reader noted that the stitch artefacts in the R-PBS images appeared more pronounced and artificial, possibly due to the use of forward projection from SPECT/CT data. Despite the increased presence of artefacts in the R-PBS images, it was determined that they were not clinically significant and would not affect report interpretation, given the availability of SPECT/CT imaging. The image preference results demonstrated no substantial difference in preference between PBS and R-PBS.

**Figure 8:**
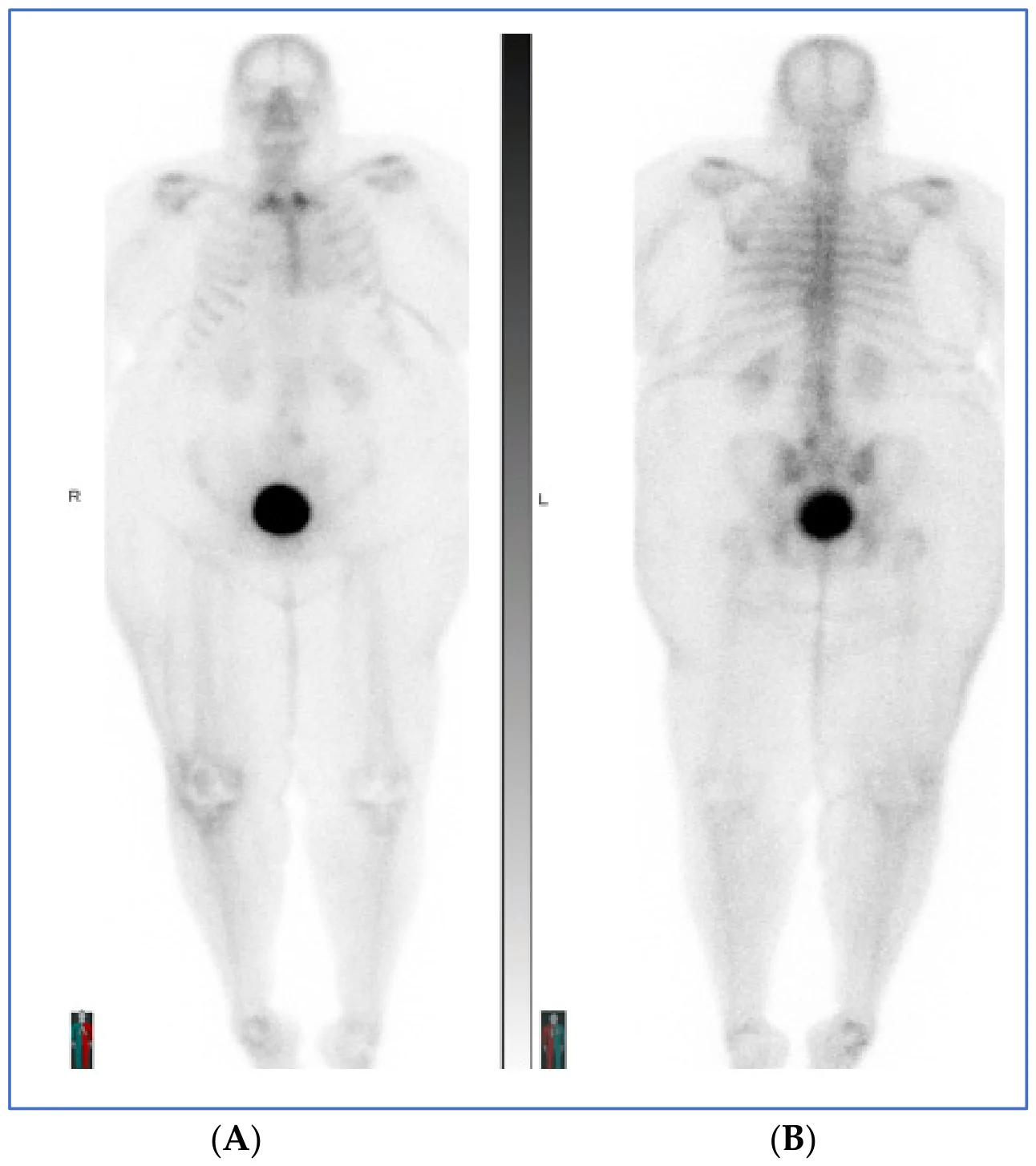
Shows the anterior (**A**) and posterior (**B**) views of PBS images.

In this study, readers commented on the observed degenerative changes in the lower extremities, outside of the field of view, in some of the study participants. In two cases, total knee replacements were reported (Figure 8). The three reading specialists determined that these were incidental findings and would not have significantly affected the final diagnosis. However, it may be advisable to consider utilising planar scanning of the lower extremities in cases where clinical symptoms, such as pain or functional impairment in the lower limbs, are present. A previous study suggested the implementation of this protocol in high-risk prostate cancer patients, which allows for immediate evaluation of images by a physician without the need for repositioning the patient and reduces the waiting time and length of stay in the imaging department [48]. Therefore, further clinical studies with a larger sample size and a variety of different diseases should be conducted to evaluate the impact of this workflow on the reporting of lower extremity conditions.

An advantage of using R-PBS in clinical settings is that it allows for the creation of 2D images without requiring extra time for planar acquisition and enables the creation of multiple planar views through a full 360° rotation. This allows for a cine of planar reprojections which may offer a more clinically familiar representation of tracer distribution compared to maximum intensity projection (MIP) images [21,26]. This study found that ~30% R-PBS images were judged to have a superior lesion-to-background ratio, although diagnostic accuracy was not assessed in this study. However, it is essential to note that further clinical research is necessary to confirm these findings and determine the overall utility of R-PBS in lesion detection. 

Beyond the oncological context evaluated here, R-PBS could extend the clinical reach of a fast WB-SPECT/CT workflow into non-oncological bone imaging. Ali et al. [16] described the diagnostic potential of ^99m^Tc-radiolabelled targeting vectors with SPECT/CT in rheumatoid arthritis and other degenerative bone and joint diseases, where earlier localisation of synovial, periarticular, and metabolic bone pathology than planar scintigraphy alone has been reported. Pairing R-PBS with 3D WB-SPECT/CT would preserve the familiar 2D overview for the reporting physician while retaining the volumetric and quantitative data required for radiomic analysis and lesion characterisation of the type demonstrated in preclinical PET radiomic studies [17], where atlas-based co-registration and extraction of a large number of radiomic features (112 per organ in the cited work) have provided both morphological and functional information. Prospective evaluation of R-PBS in non-oncological bone disease and in combination with quantitative SPECT/CT radiomics may be worth exploring in future work.

It is anticipated that there will be a shift towards the use of WB-SPECT/CT in NM for bone imaging in the future [8,49]. However, a significant challenge that has been identified with the implementation of WB-SPECT/CT is the prolonged acquisition time. According to current guidelines, the typical acquisition time for WB bone SPECT/CT imaging is approximately 40 min when accounting for detector and bed movements [5,28,50]. Also, PBS acquisition typically takes approximately 20 min. The total acquisition time would be excessively long, potentially negatively affecting workflow and patient comfort, leading to motion during imaging. However, recent studies have shown that the acquisition time for SPECT/CT can be reduced to 24 min without compromising image quality [10,27]. Zacho et al. [29] also demonstrated the feasibility of performing fast SPECT/CT as an adjunct to PBS. Additionally, next-generation SPECT-only systems using cadmium-zinc-telluride (CZT) have shown promising results in acquiring WB-SPECT/CT in less than 20 min, as reported by Gregoire et al. [30] and Arvola et al. [44]. These developments suggest that fast WB-SPECT/CT combined with R-PBS could become clinically practical, although this remains to be confirmed in larger, multicentre studies. 

The interpretation of 3D SPECT/CT images requires additional physician reading time compared to planar WB images, which may present a practical limitation that could slow the adoption of WB SPECT/CT in locations with a shortage of experienced NM physicians. The present study did not investigate this aspect of WB-SPECT/CT. Therefore, further research is necessary to understand the practical implications of the increased physician reading time associated with interpreting WB-SPECT/CT images.

This clinical study has several limitations due to time and access constraints. One of these limitations is the small number of patients involved in the study. Also, the number of different reconstruction parameter combinations was limited to four and guided by current clinical protocols rather than an extensive optimisation study design. Additionally, the study was conducted at a single centre and used a single vendor for R-PBS generation, so it would be beneficial to compare results from multiple vendors’ software in the future. Reader familiarity bias with R-PBS may have affected results. Finally, while this study was designed to evaluate the visual image quality of R-PBS, further studies will be needed to measure its diagnostic accuracy.

The principal advantages of R-PBS are: (i) elimination of a separate 15–20 min planar acquisition, with a corresponding reduction in total examination time; (ii) availability of a familiar 2D projection for interpreting physicians during the transition from PBS- to SPECT/CT-based reporting; and (iii) the ability to generate multiple reprojection angles and a cine of planar reprojections through full 360° rotation. The principal limitations are: (i) a higher frequency of artefacts, particularly edge/truncation artefacts and stitch artefacts at bed junctions; (ii) dependence on the SPECT reconstruction parameters chosen, which were not systematically optimised in this study; (iii) a lower-limb cut-off at the mid-thigh in the standard three-bed WB-SPECT/CT protocol, which may require supplementary planar imaging in cases with suspected distal pathology; and (iv) limited evidence of diagnostic equivalence, as the present study was powered for visual-quality comparison rather than lesion-level diagnostic accuracy.

## 5. Conclusions

This prospective feasibility study demonstrated that R-PBS images of acceptable to excellent quality can be generated from fast three-bed WB-SPECT/CT data using forward projection. Among the four reconstruction protocols evaluated, the combination of 6 iterations, 8 subsets, and a 5 mm Gaussian post-filter achieved the highest image quality scores and was selected for subsequent analysis. Comparison of PBS and R-PBS images revealed comparable reader assessments of image quality, image preference, and clinical significance of artefacts. These findings suggest R-PBS derived from fast WB-SPECT/CT may provide a practical approach to reduce patient imaging time by eliminating the need for a separate PBS acquisition. However, the results should be interpreted in the context of the small patient cohort and single-centre design. Large multicentre studies with formal diagnostic accuracy assessment are required before R-PBS can be considered a replacement for conventional PBS in routine clinical practice.

## Figures and Tables

**Table 1:** SPECT reconstruction parameter sets evaluated in Phase 1 for R-PBS generation.

Reconstruction No.	Iterations–Subsets	Gaussian Filter (FWHM)
1	4i8s	8 mm
2	4i8s	4 mm
3	6i8s	5 mm
4	10i5s	4 mm

**Table 2:** Participant demographics and clinical characteristics (*n* = 11).

Participant ID	Age at Scan (Years)	Sex	Clinical Indication	Relevant History or Disease Characteristics
P01	55	Female	Determine the timing of vertebral fractures	T11–L4 fractures identified on CT
P02	79	Female	Evaluate a possible new vertebral fracture	Acute thoracic back pain
P03	89	Female	Assess new or ongoing fracture changes	Recent fall
P04	87	Male	Investigate possible discitis or malignancy in the lumbar region	History of prostate cancer
P05	74	Male	Identify a source of infection using bone/gallium imaging	Underlying diagnosis and infection site not specified
P06	84	Male	Investigate possible skeletal metastatic disease	Previous breast cancer; rib pain; no fracture identified on CT
P07	57	Male	Identify occult sites of infection	Previous right toe osteomyelitis; left olecranon wound; epidural abscess and back pain
P08	80	Male	Breast cancer staging	Biopsy-confirmed right-sided invasive ductal carcinoma
P09	74	Female	Investigate possible endocarditis or spinal osteomyelitis	Staphylococcal bacteraemia
P10	74	Female	Evaluate osteomyelitis	Anatomical site and disease characteristics not specified
P11	62	Male	Investigate suspected skeletal metastases	Breast cancer

**Table 3:** Distribution of image quality scores for three readers.

Reconstruction	Score 1	Score 2	Score 3	Score 4	Good/Excellent (3–4)
4i8s, 8 mm	4	7	9	4	13/24 (54%)
4i8s, 4 mm	0 *	3	10	11	21/24 (87%)
6i8s, 5 mm	0 *	1	5	18	23/24 (96%)
10i5s, 4 mm	0 *	4	13	7	20/24 (83%)

Reconstruction: i iterations, s subsets, and FWHM Gaussian filter in mm. Visual scoring scale: 1 = unacceptable, 2 = acceptable, 3 = good, 4 = excellent. * No images were assigned score 1 (unacceptable).

**Table 4:** Median image quality scores for three readers per 8 evaluations.

Reader		“4 Iterations, 8 Subsets and 8 mm FWHM Gaussian Filter”	“4 Iterations, 8 Subsets and 4 mm FWHM Gaussian Filter”	“6 Iterations, 8 Subsets and 5 mm FWHM Gaussian Filter”	“10 iterations 5 Subsets and 4 mm FWHM Gaussian Filter”
Median Image Quality Scores:	1	3	3.5	4	3
	2	3	4	4	3
	3	2	3	4	2

Overall image quality: Planar bone scintigraphy and reprojected planar bone images.

**Table 5:** The median and interquartile range (IQR) of image quality scores, assessed by each individual reader using a 4-point ordinal visual scoring scale and the *p* values for comparison were also calculated for each reviewer.

Readers	PBS	R-PBS	PBS vs. R-PBS
	Median (IQR)	Median (IQR)	Wilcoxon Signed-Rank *p* Value
1	3 (3–4)	3 (3–4)	1.00
2	4 (4–4)	4 (2–4)	0.109
3	3 (2–3)	2 (2–3)	0.727

The median (IQR) for 11 patients, with a total of 22 images assessed for both the PBS and R-PBS.

**Table 6:** Interobserver agreement was calculated for both PBS and R-PBS by comparing the assessments of readers 1 and 2.

		Reader 2			
		Acceptable	Good	Excellent	Total
Reader 1	Acceptable	2 ^a^	0	1	3
	Good	0	6 ^a^	6	12
	Excellent	1	0	6 ^a^	7
	Total	3	6	13	22

^a^ Agreement between readers (concordant ratings); all other cells represent disagreement. No images were assigned a score of 1 (unacceptable) by any reader.

**Table 7:** Interobserver agreement was calculated for both PBS and R-PBS by comparing the assessments of readers 1 and 3.

		Reader 3			
		Acceptable	Good	Excellent	Total
Reader 1	Acceptable	3 ^a^	0	0	3
	Good	0	10 ^a^	2	12
	Excellent	4	0	3 ^a^	7
	Total	7	10	5	22

^a^ Agreement between readers (concordant ratings); all other cells represent disagreement. No images were assigned a score of 1 (unacceptable) by any reader.

**Table 8:** Interobserver agreement was calculated for both PBS and R-PBS by comparing the assessments of readers 2 and 3.

		Reader 3			
		Acceptable	Good	Excellent	Total
Reader 2	Acceptable	3 ^a^	0	0	3
	Good	0	6 ^a^	0	6
	Excellent	4	4	5 ^a^	13
	Total	7	10	5	22

^a^ Agreement between readers (concordant ratings); all other cells represent disagreement. No images were assigned a score of 1 (unacceptable) by any reader.

**Table 9:** Frequency of artefact presentations for PBS and R-PBS, as reported by three readers.

		PBS ‘Yes’/‘No’	R-PBS ‘Yes’/‘No’	Total ’Yes’/‘No’
	Reader 1	3/8	7/4	10/12
	Reader 2	3/8	8/3	11/11
	Reader 3	2/9	7/4	9/13
	Total	8/25	22/11	30/36

**Table 10:** The frequent reason for the readers’ preferences.

Superior Lesion to Background		More Number of Lesions Identified		Better Localisation		Increase Degree of Confidence	
PBS	R-PBS	PBS	R-PBS	PBS	R-PBS	PBS	R-PBS
5	9	0	1	0	0	13	8

## Data Availability

The data presented in this study are available on request from the corresponding author due to patient privacy and ethical restrictions.
